# The *GIS2* Gene Is Repressed by a Zinc-Regulated Bicistronic RNA in *Saccharomyces cerevisiae*

**DOI:** 10.3390/genes9090462

**Published:** 2018-09-19

**Authors:** Janet Taggart, Yirong Wang, Erin Weisenhorn, Colin W. MacDiarmid, Jason Russell, Joshua J. Coon, David J. Eide

**Affiliations:** 1 Department of Nutritional Sciences, University of Wisconsin-Madison, Madison, WI 53706, USA; steffen@nutrisci.wisc.edu (J.T.); wang973@wisc.edu (Y.W.); macdiarmid@nutrisci.wisc.edu (C.W.M.); 2 Department of Biomolecular Chemistry, University of Wisconsin-Madison, Madison, WI 53706, USA; erinweisenhorn@gmail.com (E.W.); jcoon@chem.wisc.edu (J.J.C.); 3 Department of Chemistry, University of Wisconsin-Madison, Madison, WI 53706, USA; 4 Morgridge Institute for Research, Madison, WI 53706, USA; jrussell@morgridge.org; 5 Genome Center of Wisconsin, Madison, WI 53706, USA

**Keywords:** zinc, homeostasis, bicistronic, RNA, transcription, regulation, gene expression

## Abstract

Zinc homeostasis is essential for all organisms. The Zap1 transcriptional activator regulates these processes in the yeast *Saccharomyces cerevisiae*. During zinc deficiency, Zap1 increases expression of zinc transporters and proteins involved in adapting to the stress of zinc deficiency. Transcriptional activation by Zap1 can also repress expression of some genes, e.g., *RTC4*. In zinc-replete cells, *RTC4* mRNA is produced with a short transcript leader that is efficiently translated. During deficiency, Zap1-dependent expression of an RNA with a longer transcript leader represses the *RTC4* promoter. This long leader transcript (LLT) is not translated due to the presence of small open reading frames upstream of the *RTC4* coding region. In this study, we show that the *RTC4* LLT RNA also plays a second function, i.e., repression of the adjacent *GIS2* gene. In generating the LLT transcript, RNA polymerase II transcribes *RTC4* through the *GIS2* promoter. Production of the LLT RNA correlates with the decreased expression of *GIS2* mRNA and mutations that prevent synthesis of the LLT RNA or terminate it before the *GIS2* promoter renders *GIS2* mRNA expression and Gis2 protein accumulation constitutive. Thus, we have discovered an unusual regulatory mechanism that uses a bicistronic RNA to control two genes simultaneously.

## 1. Introduction

In prokaryotic organisms, there are many examples where the expression of multiple genes is controlled by a single regulatory region. The classic bacterial operon comprises two or more protein-coding regions (i.e., cistrons) that are regulated by a single upstream promoter. Production of polycistronic mRNA allows for the co-regulated production of multiple, often functionally related proteins because prokaryotic ribosomes are capable of internal translation initiation. While it has been often suggested that mechanisms of multigene regulation also exist in eukaryotes, specific examples are much less common. One mechanism of multigene regulation involves genes that are divergently transcribed and contain a common promoter region between them that controls both genes. Regulation of the *GAL1* and *GAL10* genes by Gal4 in *Saccharomyces cerevisiae* is one such example [1]. This and other bidirectional promoters generate a large number of RNA species in this yeast [2] and adjacent positioning of co-regulated genes has been observed widely across eukaryotes [3]. Another mechanism found in eukaryotes that is more similar to bacterial operons is where two or more genes are transcribed in the same direction to generate bicistronic or polycistronic RNA. The presence of internal ribosome entry sites (IRES) between each cistron allows for their independent translation. A recent review identified several mammalian gene pairs for which there was evidence of polycistronic expression via IRESs [4]. In this report, we describe the discovery of a novel alternative mechanism, a bicistronic regulatory RNA that represses the expression of the two genes that it spans. Expression of this bicistronic RNA is dependent on the zinc-responsive Zap1 transcription factor.

Zinc is an essential nutrient for all organisms because of the many important roles it plays as a catalytic and structural cofactor. Thus, under zinc-deficient conditions, cells must respond to maintain intracellular zinc homeostasis. General mechanisms of zinc homeostasis include the regulation of zinc uptake, zinc efflux, compartmentalization in intracellular organelles, and the accumulation of zinc-binding proteins and small molecules that buffer zinc availability. An additional mechanism of zinc homeostasis, called zinc sparing is where specific zinc-requiring proteins are down-regulated to reduce the overall cellular demand for this nutrient [5]. 

Zinc homeostasis in the yeast *S. cerevisiae* is regulated by the Zap1 transcription factor [6]. Under zinc-replete conditions, Zap1 is not active. During zinc deficiency, Zap1 binds to DNA sequence elements called zinc-responsive elements (ZREs) in its target gene promoters and activates their transcription. The consensus sequence for a ZRE is ACCTTNAAGGT. Zap1 is thought to regulate the expression of over 80 genes. In most cases, activation of transcription by Zap1 causes increased mRNA and protein production. For example, the levels of the Zrt1, Zrt3, and Fet4 metal transporters are increased during zinc deficiency by Zap1 to facilitate zinc uptake and mobilize zinc stored in the vacuole. In contrast, Zap1-mediated expression of a noncoding intergenic transcript represses the promoter of the *ADH1* and *ADH3* genes thereby shutting off expression of these highly abundant zinc-dependent alcohol dehydrogenases [7].

In another unusual example of gene repression by the Zap1 activator protein, we recently reported on the regulation of the *RTC4* gene [8]. In zinc-replete cells, *RTC4* is transcribed from a Zap1-independent proximal promoter to produce an mRNA with a short 38 nucleotide transcript leader that is efficiently translated. Under zinc-deficient conditions, Zap1 binds upstream of the proximal promoter and induces expression of a more abundant transcript with a longer 261 nucleotides transcript leader. We refer to these transcripts as SLT and LLT for short and long leader transcripts, respectively. Production of the LLT RNA shuts off expression of the SLT mRNA likely due to physical occlusion of the promoter and/or introduction of repressive chromatin modifications in the *RTC4* proximal promoter [9]. The full *RTC4* open reading frame (ORF) is encoded by the LLT RNA but it is not efficiently translated due to the presence of four small ORFs in the transcript leader. Thus, despite the > 10-fold increase in *RTC4*-containing RNA that occurs in zinc deficiency, Rtc4 protein abundance decreases more than 10-fold. In this report, we add new insight to this story of *RTC4* regulation. The LLT RNA is bicistronic and contains the adjacent *GIS2* promoter region and ORF. We show here that LLT RNA production not only represses the *RTC4* promoter but also represses the promoter of the *GIS2* gene. To our knowledge, this is a novel mechanism of gene regulation. The widespread abundance of bicistronic and polycistronic RNAs in yeast and other eukaryotes suggest that this may be a more common mechanism for multigene regulation than is currently recognized.

## 2. Materials and Methods

### 2.1. Strains and Growth Conditions

Yeast strains were grown in rich (YPD), synthetic defined (SD), or low zinc medium (LZM), as previously described [10]. LZM contains 20 mM citrate and 1 mM EDTA to buffer pH and zinc availability. Glucose (2%) was the carbon source for all experiments. LZM + 1 µM ZnCl_2_ was routinely used as the zinc-deficient condition, and LZM + 100 µM ZnCl_2_ as the zinc-replete condition. The yeast strains used in this work were BY4743 (*MATa/MATα his3/his3 leu2/leu2 met15/MET15 lys2/LYS2 ura3/ura3*) (Thermo Fisher Scientific, Waltham, MA USA) [11], BY4743 *gis2*∆:*:KanMX/gis2*∆:*:KanMX*), BY4743 *rtc4*∆:*:KanMX/ rtc4*∆:*:KanMX*, BY4743 *tsa1*∆:*:KanMX/ tsa1*∆:*:KanMX*, BY4743 green fluorescent protein (GFP) [12], BY4741 (*MATa his3 leu2 met15 ura3*), BY4741 *GIS2*::GFP (Thermo Fisher Scientific, Waltham, MA, USA) [13], and BY4741 *GIS2:*:GFP *rtc4*∆:*:KanMX*.

### 2.2. Plasmid Constructs

To generate pRHGM and pRHGM^mZRE^, five tandem myc epitope tags were amplified from the pFL44-S-MNR2-MYC plasmid [14] with flanking regions homologous to the 3′ end of the *GIS2* ORF and its terminator and inserted into pRTC4-HA or pRTC4-HA^mZRE^ [8] by homologous recombination [15]. To generate pRG^mZRE^, pRHGM^mZRE^ was digested with *Nhe*I and *Pac*I restriction enzymes and the corresponding region, lacking the HA and myc epitope tags, was amplified from the chromosome and inserted by homologous recombination. To generate pRHGM^Ter^, the TEF terminator fragment was amplified from pFA6-KanMX4 [16] with flanking homology to the 3′ end of the *RTC4* ORF and its terminator and inserted into pRHGM by homologous recombination. To generate pGM, the *GIS2* gene was amplified from pRHGM with 500 bp upstream and 1331 bp downstream of DNA flanking the *GIS2* ORF and inserted into pRS315 [17] by homologous recombination. pGM retains the HA tags at the 3′ end of the truncated *RTC4* ORF. 

### 2.3. Quantitative RT-PCR Analysis

Quantitative analysis of gene expression by real-time polymerase chain reaction (RT-PCR) was performed as previously described using an ABI Prism 7000 (Thermo Fisher Scientific, Waltham, MA USA) [18]. The sequences of the primer pairs used were (5′-3′): *ZRT3*: TGAGCGTTACTGAGGGTTC and GTGCCTGAGCTATGGGACTG, *GIS2:* ATGTCTCAAAAAG-CTTGTTACGTTTG and GTTTGAACGTGACCGGGTTTG, *RTC4*: GCACTCACGCACAATCAGC and AAGACGTAAGGGTTTCTGGAGC, *CMD1*: TTGGCTCTGATGTCTCGTCAA and GGCG-GAGATTAAACCATCACC, *TAF10*: GGCGTGCAGCAGATTTCACAA and ACCGTCAGAA-CAACTTTGCTT, and *ACT1*: CCAGAAGCTTTGTTCCATCC and ACGGACATCGACATCACACT. Gene expression was calculated relative to the average C_t_ values for three control genes (*TAF10*, *ACT1* and *CMD1*).

### 2.4. Northern Blot Analysis

Northern blotting was performed as previously described [18]. To make strand-specific antisense probes, PCR-generated fragments were amplified from template DNA introducing the T7 RNA polymerase promoter into the products in antisense orientation by inclusion in the downstream primer. Full probe primer sequences (5′-3′) were *GIS2*: CGGTCACGTTCAAACGGA and agttaatacgactcactatagggaCTAATTCCGTCCTCTTTCATACAGTC and *TAF10*: ATGGATTTTGAGGAAGATTAC and agttaatacgactcactatagggaCTAACGATAAA-AGTCTGGGCG. Primers specific to the epitope tags were: HA: GGATCCGCTGGCTCCGCT and agttaatacgactcactatagggaTTAAGCGTAATCTGGAACGTCATATGGA, and myc: taaagctatgGAG-CAAAAGCTC and agttaatacgactcactatagggaatgTCGACAAGGCCTTGAATT. The common T7 promoter portion of each primer pair is indicated by lower case letters.

### 2.5. Protein Extraction and Immunoblotting

Yeast protein extracts were prepared for immunoblotting with a trichloroacetic acid (TCA) extraction protocol as previously described [19]. Sodium dodecyl sulfate polyacrylamide gel electrophoresis (SDS-PAGE) and immunoblotting was conducted using a Li-Cor Odyssey infrared dye detection system (LI-COR Biosciences, Lincoln, NE, USA) as previously described [19]. Antibodies used were anti-HA (12CA5, Roche product 11583816001, Sigma Aldrich, St. Louis, MO, USA), anti-myc (9e10, Invitrogen product MA1-980, Thermo Fisher Scientific, Waltham, MA, USA), anti-GFP (Roche product 11814460001, Sigma Aldrich, St. Louis, MO, USA) and anti-Pgk1 (Ab113687, Abcam, Cambridge, UK). IR 680 dye-labeled secondary anti-mouse antibody (product 680LT, lot # C30605-02) was obtained from LI-COR Biosciences, Lincoln, NE, USA.

### 2.6. Mass Spectrometry Analysis

Frozen yeast pellets were resuspended in 100 μL 6 M guanidine-HCl, 100 mM Tris pH 8 and boiled for 5 min at 100 °C. Methanol was added to a concentration of 90% to precipitate protein and the sample was centrifuged for 10 min at 14,000× *g*. The protein pellet was resuspended in 200 µL lysis buffer (8 M urea, 100 mM Tris, 20 mM TCEP, 80 mM chloroacetamide) and diluted with 1 mL 100 mM Tris pH 8. Protein digestion was performed overnight with trypsin (4 µg) before centrifuging for 5 minutes at 10,000× *g*. The resulting supernatant was de-salted with Strata C18 solid phase extraction cartridges and quantified using Pierce Quantitative Colorimetric Peptide Assay (Thermo Fisher Scientific, Waltham, MA USA). Peptides were then dried in a vacuum centrifuge before resuspending in 0.2% formic acid. 

Samples were analyzed using a liquid chromatography–mass spectrometry (LC/MS) instrument comprising an Orbitrap Fusion Lumos tribrid mass spectrometer (Thermo Fisher Scientific, Waltham, MA USA). Mobile phase A consisted of 0.2% formic acid in water and mobile phase B consisted of 0.2% formic acid in acetonitrile. A 75-min gradient ranging from 0% to 55% B was employed spanning a total runtime of 90 min. Analytes were injected onto a 1.7-micron C18 column packed in-house to a length of 35 cm and heated to 60 °C. Survey scans of peptide precursors were collected from 350–1350 Th with an AGC target of 1,000,000 and a resolution of 240,000 in the orbitrap followed by Higher-energy collisional dissociation (HCD) tandem mass spectrometry (MS/MS) turbo scans taken in the ion trap. 

The resulting LC-MS proteomic data were processed using Maxquant software version 1.5.2.8 [20] and searched against a *S. cerevisiae* database downloaded from Uniprot on 8/10/16 [21]. The digestion enzyme was set to trypsin with up to two missed cleavages, carbamidomethylation of cysteines as a fixed modification, and oxidation of methionines and protein N-terminal acetylation as variable modifications. The match between runs feature was used to decrease missing data values within the data set. Precursor mass tolerance was 20 ppm and product ions were searched at 4.5 ppm tolerances. Peptides were filtered to a 1% false discovery rate (FDR) and combined to protein groups based on the rules of parsimony. The resulting label free quantitation (LFQ) values were log2 transformed and fold changes were calculated for each condition. A Student’s *t*-test was used to calculate *p*-values, and these were converted to *q*-values to correct for multiple hypothesis testing. Statistically significant effects were defined as those proteins with 2-fold or greater changes and a *q*-value of < 0.05 after Benjamini-Hochberg correction.

### 2.7. Competitive Growth Assays

Growth assays were carried out as previously described [18]. Wild-type cells expressing GFP and isogenic untagged *gis2*∆ mutants were grown separately in liquid culture, mixed in equal numbers, and grown for 15 generations in zinc-replete or deficient media. Alternatively, wild-type cells expressing GFP and transformed with the plasmid vector and untagged wild-type cells transformed with the vector (pRS315) or pRG^mZRE^ were grown separately in liquid culture, mixed in equal numbers, and grown for 15 generations in zinc-replete or deficient media. After analysis by flow cytometry on a FACSCalibur flow cytometer (BD Biosciences, Franklin Lakes, NJ, USA), the final percentages of untagged cells in each culture were determined. A ratio below one indicated a zinc-limited growth defect. Statistical significance was determined by Student’s *t*-test after Benjamini-Hochberg correction. 

## 3. Results

### 3.1. Opposite Regulation of GIS2 mRNA and Gis2 Protein Levels in Response to Zinc

We recently completed an analysis of the effect of zinc deficiency on zinc-binding proteins of *S. cerevisiae* [22]. Of the 582 predicted zinc-binding proteins in yeast, 229 were detectable by mass spectrometry in that study. Of those, ~30 increased in abundance during the transition to zinc deficiency while ~60 decreased. To evaluate the role of transcriptional regulation in these effects, we compared the proteomics results with RNA-sequencing (RNA-seq) data from cells grown under the same zinc-replete and deficient conditions [23]. While the observed changes in the level of many proteins correlated with changes in their mRNA levels, several others did not. One of the outliers in this analysis was the *GIS2* gene and its protein product; we observed an ~2-fold increase in RNA and a 4-fold decrease in protein abundance during deficiency. *GIS2* encodes a ribosome-associated RNA-binding protein that has been proposed to facilitate translation of specific mRNAs that contain multiple repeats of the G-A/U-A/U sequence [24]. Gis2 contains seven consecutive CCHC zinc knuckle domains with the sequence C-X_2_-C-X_4_-H-X_4_-C (C = Cys, H = His, X = any amino acid). 

**Figure 1:**
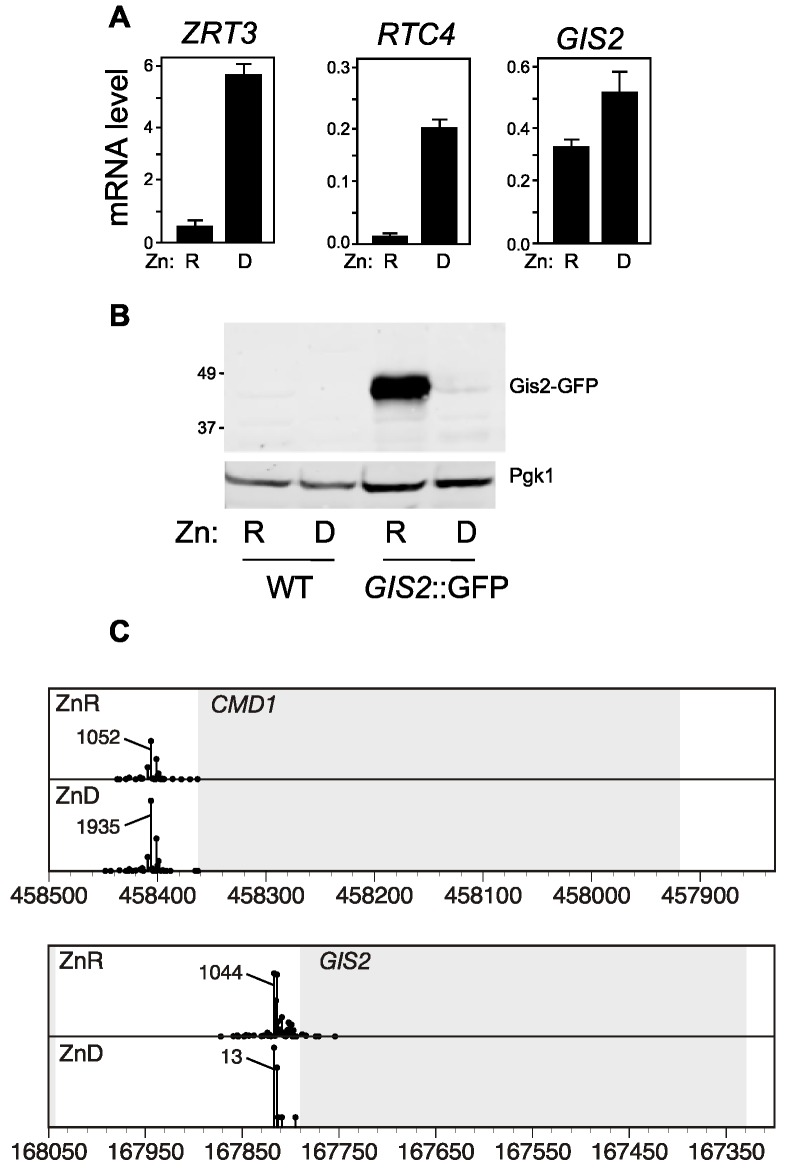
Effect of zinc status on *GIS2* mRNA and protein levels. (**A**) real time polymerase chain reaction (RT-PCR) analysis of wild-type cells (BY4741) grown in either zinc-replete (R, low zinc medium (LZM) + 100 µM ZnCl_2_) or deficient (D, LZM + 1 µM ZnCl_2_) media. The data are the means of three biological replicates and the error bars indicate ± 1 SD. All changes in response to zinc status were found to be statistically significant with *p*-values of 0.004, 0.007, and 0.035 for *ZRT3*, *RTC4*, and *GIS2*, respectively. (**B**) Immunoblot analysis of wild-type (BY4741) and BY4741 *GIS2*::green flourecent protein (GFP) cells grown under the same conditions. Anti-GFP and anti-Pgk1 antibodies were used for protein detection. The location of size markers is indicated on the left side in kDa. (**C**) Transcription start sites mapped by Deep-RACE for *CMD1* and *GIS2* under zinc-replete (ZnR) and zinc-deficient (ZnD) growth conditions are shown relative to each gene’s protein-coding region (shaded in *gray*). Independent sequencing reads representing mRNA 5′ ends are plotted across each region. The x-axis coordinates represent the position on the corresponding chromosomes. Peak values of the number of sequence reads for each gene and growth condition are shown. The transcription start site mapping data are from Wu et al. [25].

To verify these results, we used a GFP-tagged chromosomal allele of *GIS2* and RT-PCR and immunoblotting to assess mRNA and protein levels, respectively. mRNA levels of the known Zap1-regulated gene *ZRT3* increased ~12-fold in zinc-deficient cells thereby confirming the zinc status of our growth conditions, Figure 1A.

Similarly, levels of *RTC4* mRNA, encoded by the Zap1-regulated gene immediately adjacent to *GIS2*, increased 15-fold in zinc-deficient cells. Consistent with the RNA-seq results, *GIS2* mRNA levels increased 1.5-fold. In contrast to the increase in mRNA abundance and consistent with the proteomics data, the level of Gis2-GFP protein decreased markedly in deficient cells, Figure 1B. The constitutively expressed Pgk1 protein showed little change in these samples. We had previously mapped transcriptional start sites genome-wide in zinc-replete and deficient cells [25]. While transcription start sites for a control gene, *CMD1*, were little affected by zinc status, there was a marked decrease in the number of transcript start sites mapped to the *GIS2* promoter region despite the observed increase in RNA levels, Figure 1C. These results suggested that there was a major disruption of *GIS2* promoter function in zinc-deficient cells and that *GIS2*-containing RNA were being produced from a site farther upstream of the promoter and ORF.

### 3.2. Co-Regulation of RTC4 and GIS2 by a Bicistronic Regulatory RNA

**Figure 2:**
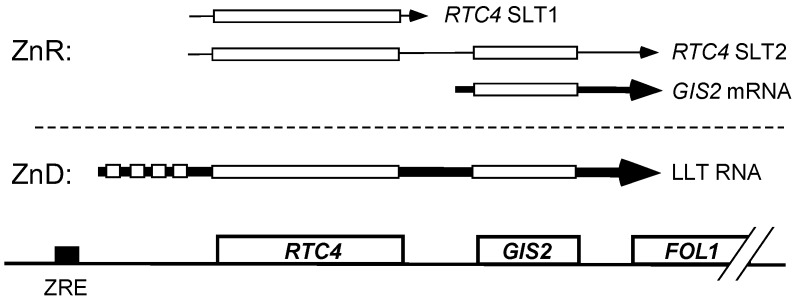
Model of *RTC4* and *GIS2* co-regulation by a bicistronic regulatory RNA. Arrows indicate RNAs produced and the boxes denote open reading frames (ORFs). Several small ORFs located in the region between the *RTC4* and *GIS2* protein-coding regions are not shown. ZnR and ZnD denote zinc-replete and zinc-deficient growth conditions, respectively. SLT1/2 and LLT refer to short leader transcripts and long leader transcripts, respectively. The box labeled ZRE indicates the location of a zinc-responsive element.

A potential explanation for these observations came from our recent studies of the *RTC4* gene [8]. *RTC4* is located immediately upstream of the *GIS2* gene, transcribed in the same direction as *GIS2*, and is regulated by Zap1 by an unusual mechanism, Figure 2. In zinc-replete conditions, *RTC4* mRNA is transcribed with a short 5′ transcript leader that is efficiently translated. Two forms of *RTC4* mRNA were detected, designated SLT1 and SLT2 for “short leader transcripts” one and two. Under zinc-deficient conditions, our studies indicated that Zap1 binds to a ZRE located 432 bp upstream of the *RTC4* ORF and activates expression of a more abundant transcript with a longer 5′ transcript leader that is poorly translated due to the presence of four small ORFs in the transcript leader. Production of this “long leader transcript” (LLT) inhibits the *RTC4* promoter and synthesis of the *RTC4* SLT1 and SLT2 mRNA likely by directly interfering with the binding of transcription factors and/or by causing formation of repressive chromatin structure. Using Northern blotting to map the endpoints of the LLT transcript, we found that it extends far beyond the *RTC4* ORF passing through the *GIS2* promoter and ORF and into the divergently transcribed *FOL1* gene [8]. Given these prior observations, we hypothesized that the bicistronic LLT transcript not only regulates *RTC4* but also controls the *GIS2* promoter by shutting off its activity in zinc-deficient cells. In contrast, because of its low level of expression, the SLT2 bicistronic transcript does not inhibit *GIS2* promoter function.

**Figure 3:**
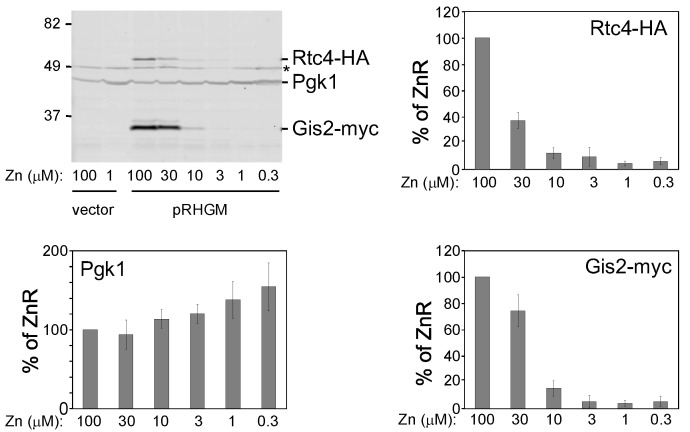
Correlation Rtc4 and Gis2 protein expression in response to zinc status. Wild-type (BY4741) cells transformed with plasmid pRHGM were grown in LZM with the indicated zinc level for 16 h prior to immunoblot analysis. pRHGM expresses HA-tagged Rtc4 and myc-tagged Gis2. Pgk1 was used as a loading control and the asterisk marks a non-specific band. The location of size markers is indicated on the left side of the panel in kDa. The histograms show quantified results from three replicates compared to 100 µM zinc cultures as the replete (ZnR) condition. Error bars are ± 1 SD.

As a first test of this hypothesis, we assessed whether Rtc4 and Gis2 protein levels were co-regulated in response to differences in zinc status. Using a plasmid expressing HA-tagged Rtc4 and myc-tagged Gis2, we found a very close correlation between the regulation of both proteins in response to zinc status, Figure 3. As zinc levels in the culture medium decreased, so were the levels of Rtc4 and Gis2 protein and with very similar dependence on zinc concentration. This correlation strongly suggested a mechanistic link regulating these two proteins.

**Figure 4:**
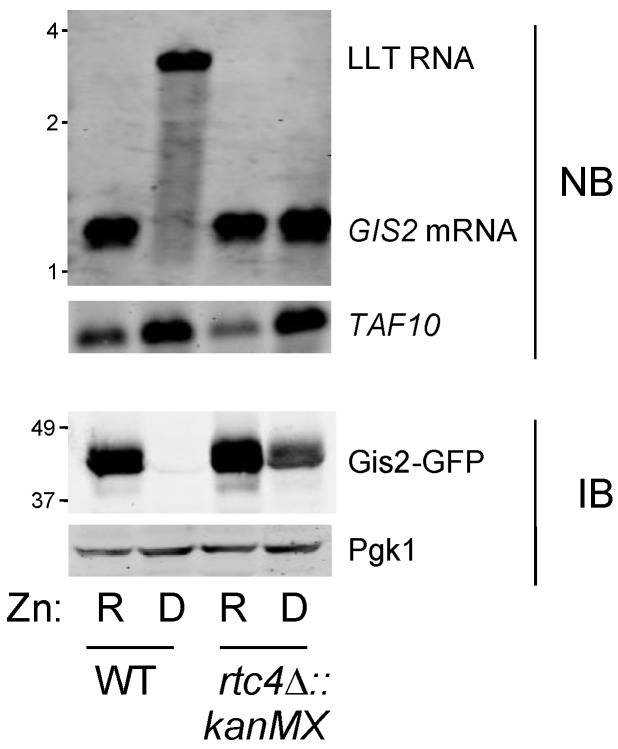
The r*tc4*∆*::kanMX* allele disrupts regulation of the adjacent *GIS2* gene. Wild-type (BY4741) and isogenic r*tc4*∆*::kanMX* mutant cells bearing a *GIS2*::GFP allele were grown in zinc-replete (R, LZM + 100 µM ZnCl_2_) or deficient (D, LZM + 1 µM ZnCl_2_) media prior to immunoblot (IB) and Northern blot (NB) analysis. Pgk1 was used as a loading control for immunoblotting and *TAF10* mRNA was used as the control for Northern blotting. The location of size markers is indicated on the left side of each panel in kb (top panel) or kDa (bottom panel).

To test the hypothesis more directly, we used an *rtc4*∆*::kanMX* mutant allele in which the entire *RTC4* ORF was replaced with the kanMX gene disruption cassette. This cassette contains a strong transcription terminator. Therefore, in addition to disrupting the *RTC4* gene, we previously demonstrated that the inserted transcription terminator blocks RNA polymerase from extending the LLT RNA beyond the cassette and into the *GIS2* promoter [8]. Thus, if repression of the *GIS2* promoter is mediated by the LLT RNA, this allele is expected to result in constitutive *GIS2* expression. Using a *GIS2*-GFP fusion allele, Northern blotting and immunoblotting indicated that the *rtc4*∆*::kanMX* allele resulted in constitutive *GIS2* mRNA and Gis2 protein expression, Figure 4. This result supported the hypothesis that the LLT transcript represses the *GIS2* promoter. A small decrease in Gis2-GFP protein was observed in zinc-deficient *rtc4*∆*::kanMX* cells perhaps because of some protein degradation.

**Figure 5:**
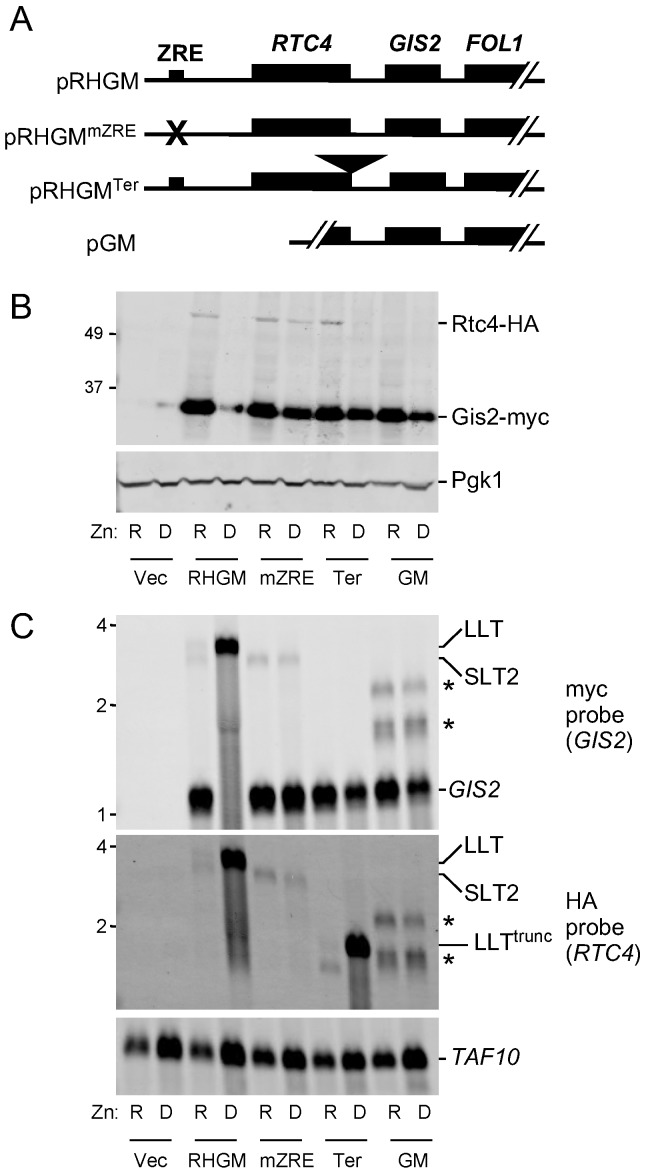
Mutagenesis analysis of *RTC4* and *GIS2* co-regulation. Wild-type (BY4741) cells were transformed with plasmids pRHGM, pRHGM^mZRE^, pRHGM^Ter^, or pGM were grown in grown in zinc-replete (R, LZM + 100 µM ZnCl_2_) or deficient (D, LZM + 1 µM ZnCl_2_) media prior to analysis. (**A**) Diagram of the plasmids used in this experiment. (**B**) Immunoblot analysis of Rtc4-HA, Gis2-myc, and Pgk1. (**C**) Northern blot analysis of RNA expression using HA tag and myc tag-specific probes. *TAF10* was used as a loading control. The locations of the *GIS2* and *RTC4* SLT2 mRNAs are marked as well as the LLT RNA and truncated LLT RNA produced in the pRHGM^Ter^ transformant. *RTC4* SLT1 was not detectable in this experiment because of its low abundance. The *asterisks* mark two transcripts that arise from transcription initiation in the plasmid vector for pGM. The location of size markers is indicated on the left side of each panel in kDa (panel B) or kb (panel C).

While the *rtc4*∆*::kanMX* allele introduces a transcriptional terminator, it also deletes a large chromosomal region upstream of *GIS2* gene. Thus, an alternative hypothesis to explain the results in Figure 4 was that this deletion/insertion disrupts elements of the *GIS2* promoter located in or near the *RTC4* ORF that are required for decreasing *GIS2* expression in zinc-deficient cells. To address this hypothesis, we generated additional mutant constructs in plasmids encoding HA-tagged Rtc4 and myc-tagged Gis2. With the wild-type version of this plasmid (pRHGM), Rtc4-HA and Gis2-myc protein levels were down-regulated in zinc-deficient cells and this coincided with production of the LLT transcript and loss of *RTC4* (SLT2) and *GIS2* mRNA expression, Figure 5B,C. SLT1 was not detectable in this experiment due to its lower abundance. With a mutant allele in which the ZRE was mutated to a sequence not bound by Zap1 (pRHGM^mZRE^, Figure 5A), no LLT RNA was produced, *RTC4* SLT2 and *GIS2* mRNAs were constitutively expressed, and Rtc4 and Gis2 proteins were also constitutively expressed. Insertion of a strong transcriptional terminator just downstream of the *RTC4* stop codon (pRHGM^Ter^) had no effect on zinc-responsive Rtc4 regulation but Gis2 protein was constitutively produced. This allele produced a truncated LLT transcript detectable with the HA tag-specific NB probe. Finally, a plasmid containing the *GIS2* promoter and 245 bp of additional sequence upstream from the *RTC4* ORF (pGM) produced no Rtc4 protein and Gis2 was constitutively expressed. These results are all consistent with both *RTC4* and *GIS2* being regulated by the same bicistronic regulatory RNA. Two new transcripts were observed with the *GIS2*-only pGM plasmid, Figure 5C, *asterisks*, and their sizes suggested that they arose from transcription initiation in the plasmid vector upstream of the insert.

### 3.3. Probing the Functional Significance of GIS2 Regulation

The function of Gis2 is not well characterized. However, several studies have suggested a role for Gis2 in controlling the accumulation of specific proteins through mechanisms affecting either translation or transcription. Therefore, we carried out an analysis of the total proteome of yeast with altered expression of Gis2 because this approach would be likely to detect effects in either transcriptional or post-transcriptional mechanisms that alter protein abundance. In one experiment, we compared wild-type vs *gis2*∆ cells grown in zinc-replete conditions. Under these conditions, we hypothesized that the abundance of proteins that were influenced by Gis2 function either positively or negatively would be altered. Second, we compared wild-type cells transformed with a plasmid vector vs. wild-type cells transformed with a plasmid (pRG^mZRE^) containing the *RTC4*-*GIS2* chromosomal region that was mutated in its ZRE thereby resulting in constitutive *RTC4* and *GIS2* expression. These cells were grown under zinc-deficient conditions where Rtc4 and Gis2 are normally repressed. We hypothesized that the abundance of any other proteins affected positively or negatively by repression of *RTC4* and *GIS2* would be detected. This study used untagged alleles of *RTC4* and *GIS2* to avoid any disruption of function that may be caused by epitope tagging. Furthermore, we used a condition of more moderate zinc deficiency (LZM + 6 µM ZnCl_2_) because under these conditions repression of Rtc4 and Gis2 is normally still strong, Figure 3, yet the level of zinc available for binding to Gis2 to confer function is higher than under more severe deficiency conditions.

**Figure 6:**
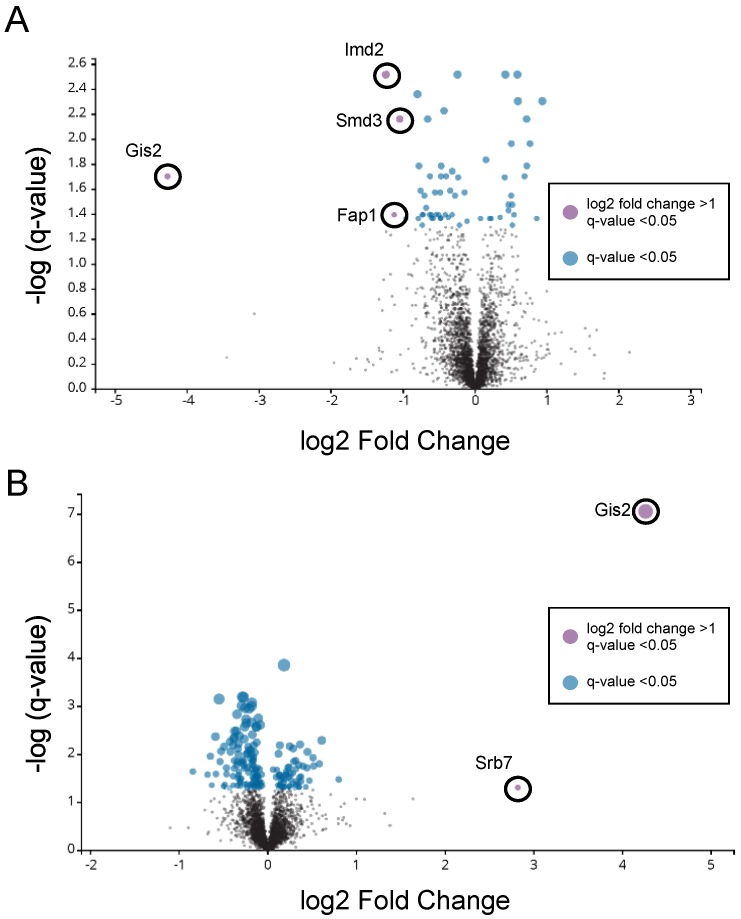
Proteomics analysis of cells with altered *GIS2* expression. Mass spectrometry was used to analyze protein abundance in, (**A**) *gis2*∆ vs. wild-type zinc-replete cells (LZM + 100 µM ZnCl_2_) and (**B**) wild-type cells transformed with either the vector (pRS315) or pRG^mZRE^ grown under zinc-deficient conditions (LZM + 6 µM ZnCl_2_). The volcano plots show the data plotted as −log (*q*-value) vs. log2 fold change. Data are based on four biological replicates for each strain/growth condition. Statistically significant effects were defined as those proteins with 2-fold (log2 = 1) or greater changes and a *q*-value of < 0.05 after Benjamini-Hochberg correction. The full data from this analysis are provided in Table S1.

Deletion of *GIS2* in replete cells resulted in decreased abundance of three proteins in addition to Gis2, i.e., Imd2, Smd3, and Fap1, Figure 6A. No proteins were observed to increase significantly in replete *gis2*∆ mutant cells. Overexpression of *RTC4* and *GIS2* in zinc-deficient cells caused an increase in only Gis2 and one other protein, Srb7; statistically significant changes were not detected for any other proteins, Figure 6B. Rtc4 was undetectable in these experiments because it is of very low abundance. Moreover, immunoblot analysis was performed with GFP-tagged alleles of four mRNA previously proposed to be direct targets of Gis2 translational control (*BDH1*, *BUD2*, *FCY1*, and *THS1*) [24]. These four proteins are down-regulated during zinc deficiency [26] but Gis2 overexpression in zinc-deficient cells had no effect on their protein abundance (data not shown). These results indicated that in our strains and under our growth conditions, Gis2 plays only a limited role in zinc-replete cells and loss of *GIS2* regulation has little effect on the proteome of zinc-deficient cells.

Finally, using a sensitive co-culturing method, we examined the effect of *gis2*∆ mutation and constitutive *RTC4*/*GIS2* expression on cell growth. Cells with altered Gis2 expression were mixed in approximately equal numbers with GFP-tagged control cells. The mixed cells were then inoculated into zinc-replete and deficient cultures and grown for 15 generations prior to measuring the abundance of GFP-tagged control cells and untagged cells by Fluorescence-activated cell sorting (FACS). In zinc-replete conditions, we confirmed previously published results [27,28] that mutation of *GIS2* caused a small but detectable decrease in fitness, Table 1. In zinc-deficient cells, mutation of *GIS2* had no detectable effect on growth rate. Cells mutant for the *TSA1* gene were included as a positive control in this experiment; the growth defect of *tsa1*∆ mutants in zinc-replete conditions that is greatly exacerbated by deficiency observed previously [18] was confirmed here. Constitutive expression of *RTC4* and *GIS2* had no effect on zinc-replete growth but, surprisingly, conferred a small growth advantage in deficient cells. Thus, Gis2 is of some importance to the growth of cells under zinc-replete conditions but overexpression in zinc-deficient cells when it is normally repressed had no negative effects on growth. 

## 4. Discussion

In a recent report, we described an unusual mechanism in which the transcriptional activator Zap1 mediates repressed translation of *RTC4* mRNA [8]. Under zinc-replete conditions, the SLT1 and SLT2 mRNA are produced from the *RTC4* gene and are efficiently translated. During zinc deficiency, Zap1 activates expression of the longer LLT RNA. This regulatory RNA shuts off expression from the *RTC4* promoter. In addition, the *RTC4* ORF contained within the LLT RNA is not efficiently translated because of four small ORFs located upstream in the transcript leader. Thus, Rtc4 protein levels decrease despite a > 10-fold elevation in RNA levels. In this report, we show that the LLT regulatory RNA may have a second function, i.e., to shut off the *GIS2* promoter. To our knowledge, this mechanism whereby a single bicistronic regulatory RNA represses two adjacent genes is unprecedented. Despite its novelty, however, similar mechanisms may function to regulate other genes in yeast as well in other organisms. Pelechano et al. discovered that 185 of 2727 (6.7%) adjacent gene pairs in yeast were expressed to at least some level as bicistronic RNAs [29]. Bicistronic RNAs have also been found in several viruses of eukaryotes, *C. elegans*, *Drosophila*, and vertebrates [4,30,31]. For many of these examples, the bicistronic RNAs have been either shown or proposed to coordinate protein expression through IRESs. IRESs are specialized RNA elements that allow for the cap-independent translation of internal ORFs in mRNA [32]. In contrast, the bicistronic LLT RNA of the *RTC4*/*GIS2* region serves a repressive regulatory role and does not produce either Rtc4 or Gis2 protein to a detectable level.

Through what mechanism does the bicistronic LLT transcript shut off the *GIS2* promoter? Possible mechanisms fall into either of two general categories, i.e., cis-acting and trans-acting mechanisms. In a trans-acting mechanism, the LLT transcript is produced and released from the DNA template after transcription and somehow inhibits the promoter, e.g., by binding to and interfering with transcription factor function. Our results argue against this general type of mechanism. As shown in Figure 5 where expression of the LLT transcript from the plasmid is disrupted (e.g., the mutant ZRE construct), the LLT transcript is still produced by the chromosomal gene yet it exerts no apparent negative impact on *GIS2* expression from the plasmid. Possible cis-acting regulatory mechanisms include the physical interference with transcription factor binding to the *GIS2* promoter by the movement of LLT-transcribing RNA polymerase II through that region of the DNA. Alternatively, it is well established that RNA polymerase II recruits chromatin-modifying proteins to transcribed regions that repress transcription from initiating within those regions [33]. This mechanism commonly inhibits the activity of cryptic promoters within genes and thereby prevents expression of truncated proteins. Either or both cis-acting mechanisms may be involved in repressing the *RTC4* and *GIS2* promoters. 

Our studies of *RTC4* and *GIS2* strongly suggest that they are directly regulated by the zinc-responsive Zap1 transcription factor. The LLT transcript is dependent on Zap1, is constitutively produced in cells with a constitutively active Zap1 allele, and the consensus Zap1 binding site upstream of the *RTC4* promoter is required for LLT expression [8], Figure 5. What is the purpose of this regulation in zinc-deficient cells? The fact that *RTC4* and *GIS2* are co-regulated by the LLT RNA suggests that they share a common function [34]. The function of the Rtc4 protein is unclear so it provides few clues to possible roles for Gis2. Rtc4 is found in both the cytosol and nucleus and genetic studies have linked it to telomere maintenance [13,35]. Studies of Gis2, however, have provided more specific hypotheses to consider. The *GIS2* gene was first identified as a high copy suppressor of a strain unable to grow on galactose-containing media because of disrupted transcription of galactose metabolism genes [36]. The Gis2 protein contains seven CCHC-type zinc knuckle domains and related proteins bind to single-stranded DNA and to RNA. Gis2 immunoprecipitation experiments combined with microarray analysis indicated that Gis2 binds to stretches of G-A/U-A/U repeats in hundreds of mRNAs [24]. Based on a combined transcriptomics/proteomics analysis, it was concluded that Gis2 reduces the levels of many mRNA to which it binds. Many of these target mRNAs are involved in cytosolic translation. Gis2 was also found in stress-induced P-bodies and stress granules where it was proposed to inhibit translation of specific mRNA during stress conditions [37]. Finally, a recent study of Gis2 orthologs in *Cryptococcus neoformans* implicated their function in the post-transcriptional control of ribosomal proteins [38].

Gis2 is the yeast ortholog of mammalian CNBP/ZNF9 and these proteins are functionally conserved; expression of CNBP/ZNF9 in yeast *gis2*∆ mutants complemented some of its phenotypes [24]. Therefore, clues to Gis2’s function may also come from the analysis of its mammalian relative. *CNBP/ZNF9* is ubiquitously expressed in adult tissues and whole-animal knockout mutations in mice have been shown to be embryonic lethal [39]. In humans, expansion of a CCTG repeat in intron 1 of *CNBP/ZNF9* causes myotonic dystrophy 1, a common type of muscular dystrophy [40]. At least some of the symptoms of this disease-causing repeat expansion are due to loss of CNBP/ZNF9 function [41]. Analysis of CNBP/ZNF9 indicated that it also binds to G-rich mRNA sequences that can form G-quadruplex structures [42]. These structures inhibit translation [43] and CNBP/ZNF9 binding prevented their formation and promoted translation of the affected mRNA. In addition to these effects, CNBP/ZNF9 has also been implicated as a DNA-binding transcriptional regulator [44]. Thus, prior studies of Gis2 and CNBP/ZNF9 suggested transcriptional or post-transcriptional roles for Gis2 in regulating the abundance of specific proteins.

In our proteomics analysis, Gis2 was implicated in affecting the expression of only three proteins in replete cells, Imd2, Smd3, and Fap1. Imd2 is involved in GTP synthesis, Smd3 is a spliceosomal subunit, and Fap1 is a transcription factor. This limited effect was surprising given the hypothesized role of Gis2 in regulating the translation of hundreds of mRNAs [24]. In addition, disrupting the mechanism that represses *GIS2* expression in zinc-deficient cells had little detectable impact on the proteome in these cells; only Srb7, a component of the Mediator complex, was affected [45]. One caveat is that our proteomics analysis only detected about half of the total yeast proteome. Nonetheless, many of the proteins previously found to be impacted by Gis2 were detectable in our analysis. Disruption of the *GIS2* gene in zinc-replete cells and constitutive expression of Gis2 protein in deficient cells had little impact on growth. Thus, we obtained no evidence indicating that repression of *GIS2* serves any broad regulatory role under the specific conditions of our experiments. It remains possible that Gis2 regulates the abundance of many of its target proteins under other growth conditions and that regulation of Gis2 activity in response to zinc status more broadly impacts protein expression under those conditions.

An alternative purpose of shutting off Gis2 expression in zinc-deficient cells is zinc sparing, i.e., the decreased expression of specific zinc-dependent proteins, as a means of reducing the cellular zinc requirement during deficiency [5]. Gis2 has been estimated to accumulate between 24,000 and 100,000 molecules per cell under replete conditions [46,47]. With seven zinc ions bound per monomer, this translates into 168,000–700,000 zinc ions required by Gis2 per cell or 2–8% of the total zinc requirement of the cell [48]. Thus, repression of Gis2 may be a mechanism that allows zinc-deficient cells to divert a limited supply of the metal to more important functions. The absence of a growth defect in zinc-deficient cells with constitutive Gis2 accumulation may then reflect the cumulative importance of multiple zinc-sparing mechanisms and the limited impact of disrupting any single such mechanism on the total zinc requirement [49].

## Figures and Tables

**Table 1:** Effect of *gis2*∆ mutation and *RTC4/GIS2* constitutive expression on cell growth.

		Zinc Replete			Zinc Deficient		
Strain	% in T_o_ Inoculum	% after 15 Generations	Exp/Cont Ratio	*p*-Value ^a^	% after 15 Generations	Exp/Cont Ratio	*p*-Value ^a^
WT	53.9	52.7 ± 0.2	-	-	49.8 ± 2.6	-	-
*gis2∆*	49.9	46.1 ± 1.4	0.9	0.02	49.9 ± 1.3	1.0	NS
*tsa1∆*	50.1	28.9 ± 1.2	0.6	0.001	1.4 ± 0.1	0.03	0.001
WT vector	51.7	49.0 ± 0.6	-	-	45.2 ± 0.6	-	-
WT pRG^mZRE^	50.9	49.5 ± 0.7	1.0	NS	53.2 ± 0.9	1.2	0.006

^a^ NS = Not significant.

## Supplementary Materials

The following are available online at http://www.mdpi.com/2073-4425/9/9/462/s1: Table S1. Results of the proteomics analysis of wild-type vs. *gis2*∆ mutant cells grown in replete zinc and vector vs. constitutive *RTC4/GIS2* expression in deficient zinc conditions.
